# An Emerging Cause of Arboviral Encephalitis in Pennsylvania

**DOI:** 10.7759/cureus.85053

**Published:** 2025-05-29

**Authors:** Chandan K Dash, Ryan Rothman, Arshpal Gill, Nitin Bhanot, Zaw Min

**Affiliations:** 1 Department of Internal Medicine, Allegheny Health Network, Pittsburgh, USA; 2 Division of Infectious Diseases, Allegheny Health Network, Pittsburgh, USA

**Keywords:** arboviral disease, encephalitis, powassan virus, powassan virus encephalitis, powv, tick-borne disease, tick borne encephalitis, tick-borne flavivirus

## Abstract

Powassan virus (POWV) is a rare neuroinvasive flavivirus transmitted through the bite of an infected *Ixodes scapularis* tick. Although infections are infrequent, they have become increasingly recognized in recent years, particularly in the northeastern United States. With the expanding geographic range of *Ixodes* ticks due to climate change, the risk of POWV transmission is rising, making awareness of the virus crucial for timely diagnosis and effective management. Preventive measures, such as avoiding tick exposure, using repellents, and performing thorough tick checks, are essential in reducing the risk of infection. Here, we present a case of POWV encephalitis in western Pennsylvania, highlighting some of the diagnostic challenges.

## Introduction

Powassan virus (POWV) is a single-stranded RNA virus that belongs to the *Flaviviridae* family. The virus is transmitted to humans through the bite of an infected *Ixodes* tick. Although multiple *Ixodes* species (*I. scapularis*, *I. cookei*, *I. marxi*, *I. spinipalpus*) have been described to harbor the virus, *I. scapularis* has been identified as the principal vector in humans. POWV is a rare but increasingly recognized cause of zoonotic neuroinvasive infection in North America. Its incidence in the United States has been steadily increasing since the early 2000s, with the highest number of cases reported in the northeastern states, followed by the upper Midwest and the Great Lakes regions. The recent increase in POWV cases can be attributed to multiple factors, including the expanding geographic range of ticks, changes in climate and ecological conditions, and improved awareness and diagnostic testing. POWV neuroinvasive infection can present with a wide range of neurologic manifestations, including headache, fever, altered mental status, seizures, and focal neurologic deficits. Owing to its nonspecific presentation and overlap with other viral and bacterial etiologies of meningoencephalitis, POWV infection often presents a broad differential diagnosis. This case report describes a POWV neuroinvasive infection in a patient from western Pennsylvania, USA. It outlines the mode of transmission, clinical presentation, diagnostic challenges, and the growing importance of POWV as a cause of encephalitis. It also emphasizes the need for increased awareness among healthcare providers and a high degree of clinical suspicion, especially in regions where *Ixodes* ticks are endemic.

## Case presentation

A 65-year-old female presented to our hospital with an insidious onset of progressive malaise, fatigue, and confusion during the mid-winter season. The patient had reportedly experienced multiple falls on the day of admission. She had dogs and cats as pets, and horses were present in the surrounding farmland. According to her family members, rodent carcasses, brought into the home by the pets, were found inside closets and dark corners of the house. The patient had reportedly sustained three tick bites on separate occasions while walking her dogs during the month prior to admission. On presentation, she was febrile to 102°F, with a heart rate of 83 beats per minute, blood pressure of 170/74 mm Hg, and respiratory rate of 18 breaths per minute. She was somnolent and unable to provide a history due to decreased level of consciousness. Given concerns about respiratory compromise, she was intubated in the ED for airway protection. Physical examination was unremarkable aside from somnolence; specifically, there was no skin rash or joint swelling. Laboratory investigations on admission were notable for neutrophilic leukocytosis, with a WBC count of 17,380/mcL (77% neutrophils), baseline renal function (serum creatinine 1.16 mg/dL, blood urea nitrogen 19 mg/dL), and normal liver function tests except for mild elevations in Aspartate Aminotransferase (AST; 54 U/L) and Alanine Aminotransferase (ALT; 44 U/L). CT of head was done which did not reveal any acute abnormality. Blood cultures were collected, and a lumbar puncture (LP) was performed. Empiric intravenous therapy was initiated with ceftriaxone, vancomycin, ampicillin, and acyclovir. Doxycycline was added until Lyme serology, Anaplasma phagocytophilum antibody, and PCR results returned negative. CSF analysis (Table 1) revealed a total WBC count of 349/mcL (normal: 0-5/mcL) with 80% neutrophils, protein level of 92 mg/dL (normal: 15-45 mg/dL), glucose of 64 mg/dL (with corresponding serum glucose of 113 mg/dL), and elevated red blood cells at 134/mcL (normal: 0/mcL). CSF Gram stain was negative, and CSF culture showed no bacterial growth. The CSF BioFire® FilmArray® meningitis/encephalitis molecular panel was also negative.

**Table 1:** Findings from serial CSF analysis. Serial CSF analysis shows a persistent elevation of RBCs, indicating ongoing inflammation. However, the nucleated cell profile demonstrates a clear shift from a neutrophil-predominant pattern on day 2 to a lymphocyte-predominant pattern on day 4, a change commonly observed in viral encephalitis. The reason for the decreased glucose level in the CSF sample on day 4 remains unclear. VDRL: Venereal Disease Research Laboratory.

Parameter	Day 2 of Hospitalization	Day 4 of Hospitalization
Color	Colorless	Colorless
Clarity	Clear	Clear
Protein (15-45 mg/dL)	92 mg/dL	<6 mg/dL
Glucose	64 mg/dL	<2 mg/dL
Lactate (1.1-2.4 mmol/L)	2.1 mmol/L	2.1 mmol/L
WBC (0-5/mcL)	713 (Tube 1)	6 (Tube 3)
	85% Neutrophils	1% Neutrophils
	12% Lymphocytes	85% Lymphocytes
	3% Monocytes	14% Monocytes
	349 (Tube 2)	2 (Tube 4)
	80% Neutrophils	4% Neutrophils
	18% Lymphocytes	90% Lymphocytes
	2% Monocytes	7% Monocytes
RBC (0/mcL)	220 (Tube 1)	131 (Tube 3)
	134 (Tube 2)	229 (Tube 4)
BioFire® FilmArray® Meningitis/Encephalitis Panel	Negative	Negative
VDRL	Negative	Not Tested
West Nile Virus IgG Antibody	Positive	Not Tested
West Nile Virus IgM Antibody	Negative	Not Tested
Cryptococcal Antigen	Negative	Not Tested
Bacterial Gram Stain and Culture	No Growth	No Growth

**Figure 1:**
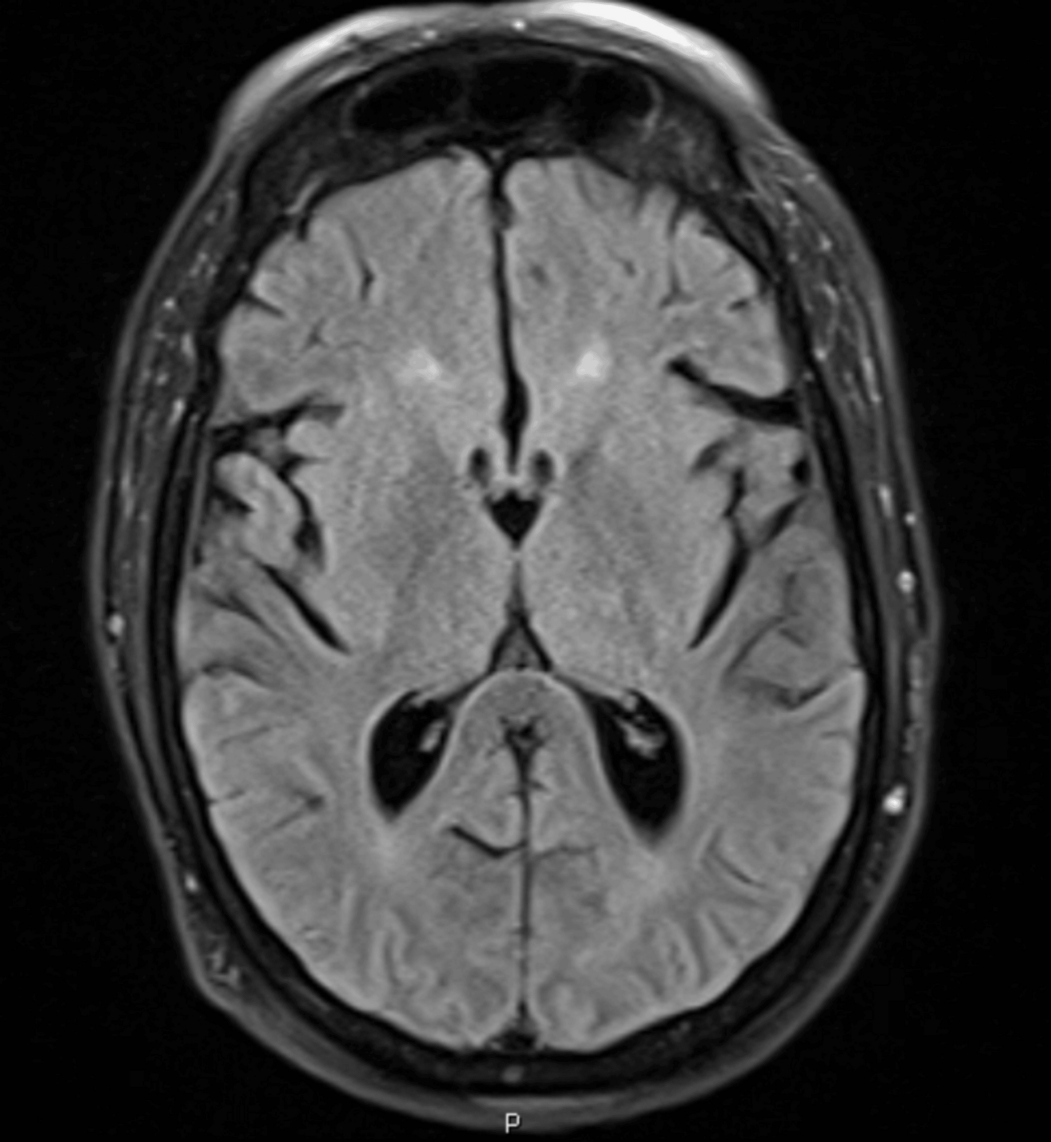
Contrast-enhanced MRI of the brain. Contrast-enhanced MRI of the brain did not reveal any parenchymal lesions or leptomeningeal enhancement.

Contrast-enhanced MRI of the brain (Figure 1) did not reveal any leptomeningeal enhancement or cerebral parenchymal lesions. EEG showed generalized theta wave slowing, suggestive of an encephalopathic process. The patient’s mental status did not improve. Due to a high RBC count in the CSF and persistent encephalopathy, a repeat LP was performed on the fourth day of hospitalization, as herpes simplex virus (HSV) encephalitis was suspected. The repeat CSF analysis (Table 1) showed a decrease in total WBC count to 6/mcL (predominantly lymphocytes), but the CSF RBC count remained elevated at 229/mcL. The CSF HSV PCR using the BioFire® FilmArray® meningitis/encephalitis panel remained negative, and intravenous acyclovir was subsequently discontinued. Given the history of rodent exposure, tularemic meningitis and lymphocytic choriomeningitis (LCM) virus encephalitis were considered, as the patient's encephalopathy persisted. Empiric intravenous gentamicin was initiated for suspected tularemic meningitis until Francisella tularensis serology returned negative. The serum LCM virus antibody panel also returned negative. Although CSF West Nile virus IgG was positive, it was considered indicative of past exposure or a false positive, as the CSF West Nile virus IgM assay was negative. All antibiotics were discontinued after both CSF cultures showed no growth. The patient's neurological status gradually improved, and she was successfully extubated. Upon extubation, she was alert and oriented to time, place, and person. She then recalled the tick bites and reported saving one of the ticks. The family brought the tick to the hospital; it was identified in the lab as an engorged female *Ixodes scapulari*s. The Pennsylvania State Health Department and the CDC were contacted for POWV serology testing. The POWV IgM enzyme-linked immunosorbent assay (ELISA) was positive, and a high POWV plaque reduction neutralizing antibody titer of 1:5120 (normal <1:10) confirmed the diagnosis of POWV encephalitis. After a prolonged hospital stay, the patient was discharged home following a brief inpatient rehabilitation. At a two-month outpatient follow-up, she was living independently and did not require assistance with activities of daily living, though she reported some residual numbness in her hands and feet and intermittent dizziness.

## Discussion

POWV is a single-stranded RNA virus that belongs to the *Flaviviridae* family, which includes other well-known viruses such as West Nile virus, dengue virus, and Zika virus [1]. POWV infection was first identified in 1958 in Powassan, Ontario, Canada, and has since been reported in parts of North America, particularly the northeastern United States and parts of Canada [2]. According to the CDC, POWV infection remains relatively rare. From 2004 to 2014, 72 cases, and from 2015 to 2023, 268 cases of POWV infection were reported to the CDC. An additional 50 cases were reported in 2024. The majority of cases in the United States have occurred in the northeastern, upper Midwest, and Great Lakes regions, areas endemic to ticks of the *Ixodes* genus. Over 20 years (2004-2024), a total of 17 cases of POWV have been reported from the state of Pennsylvania. Among them, only 2 cases were reported from western Pennsylvania [3]. However, the actual incidence of POWV infection is likely underestimated due to under-recognition among healthcare providers.

POWV infection is an arthropod-borne virus (arbovirus) transmitted by infected *Ixodes* species ticks (mainly *I. scapularis*). In southwestern Pennsylvania, the prevalence of POWV among *Ixodes* ticks is very low (<1%), in contrast to >30% with *Borrelia burgdorferi*, 10% with *Anaplasma phagocytophilum*, and 1% with *Babesia* species (communication with Pennsylvania Department of Health). The lifecycle of POWV involves its definitive animal hosts, which are mostly small mammals (such as rodents, groundhogs, and squirrels). Humans are incidental hosts and do not serve as a reservoir of infection. Ticks become infected when they feed on definitive host animals with POWV viremia. The infected ticks then bite and transmit the virus to humans [4]. Notably, person-to-person transmission of POWV through blood transfusion has been very rarely reported. Thus, individuals infected with POWV are advised not to donate blood or bone marrow for 120 days following infection [5].

Tick-borne diseases usually peak in the spring and summer seasons when ticks are most active. However, due to global warming and resulting climate change, *Ixodes* ticks may remain active during the fall and early winter. This may explain the increasing incidence of *Ixodes*-borne diseases such as Lyme disease, anaplasmosis, and POWV infections in the off-season during fall and winter [6-7].

While tick-borne diseases such as Lyme disease require an infected tick to be attached for over 24 hours to transmit the infection, studies have shown that tick attachment for as little as 15 minutes may be sufficient to transmit POWV [8]. Once introduced into the human body, POWV infects and replicates in various tissues, with a particular predilection for the CNS. The virus can cross the blood-brain barrier and enter neurons, leading to neurological complications. The mechanism by which the virus crosses this barrier is not entirely understood, but it is believed that the virus may use receptor-mediated endocytosis or direct infection of endothelial cells to gain access to the brain [9].

The clinical presentation of POWV infection can be classified into non-neuroinvasive and neuroinvasive forms. Non-neuroinvasive POWV infection presents as a febrile, flu-like illness resembling other viral infections. Clinical manifestations of neuroinvasive POWV infection include fever, headache, vomiting, confusion, loss of coordination, speech difficulty, memory loss, seizures, and changes in personality, symptoms suggestive of encephalitis. These symptoms typically develop within a week to a month after being bitten by an infected tick [10]. Approximately 23% of infected individuals develop the neuroinvasive form of infection [11].

Although the radiological findings in neuroinvasive POWV infections can be widely variable and non-specific, about 25% of patients have been reported to lack any acute abnormalities on head CT and brain MRI. When present, the most frequently observed neuroradiological abnormalities are hyperintensities on T2-weighted and fluid-attenuated inversion recovery (FLAIR) sequences, indicative of underlying inflammation and cerebral edema. The most commonly involved anatomical regions are the basal ganglia (50%), cerebellum (40%), thalamus (30%), brainstem (30%), cerebral cortex, and temporal lobes (10%). Involvement of the cerebellum is thought to be a poor prognostic factor, as it is more common in fatal cases of POWV infection (70%) compared to non-fatal cases (20%). EEG findings are likewise nonspecific, typically demonstrating generalized slowing of theta waves, consistent with an encephalopathic state. Superimposed spike-wave discharges may also be observed, often localized to regions correlating with the radiologic abnormalities described above [12]. CSF analysis, as seen in our patient, can show neutrophilic predominance in the early phase, as neutrophils are the first cells to be recruited to the site of inflammation. However, later in the disease course, lymphocytes become more predominant in the CSF, denoting a viral rather than bacterial encephalitis process [13].

The severity of neurological involvement is largely age-dependent, with older adults being at higher risk for developing severe complications. Severe disease is also more commonly seen in males, for reasons that remain unclear [14]. Approximately 50% of those who develop encephalitis from POWV infection experience long-term neurological sequelae, including cognitive dysfunction, motor impairment, and memory problems [10]. It is important to recognize POWV as one of the causes of meningoencephalitis in regions where *I. scapularis* ticks are endemic. Otherwise, extensive diagnostic testing in search of the causative agent for this debilitating neurologic infection could be conducted in vain, adding to healthcare costs and emotional burden for patients’ families.

Owing to its rarity, the key to diagnosing POWV encephalitis is a high degree of clinical suspicion in patients with unexplained encephalitis in *Ixodes* tick-endemic areas. A history of tick bite or identification of the tick, as in our patient, can help identify patients with a high pretest probability of POWV encephalitis. A positive IgM ELISA is considered the first diagnostic test. However, positive POWV IgM ELISA results should be confirmed by more specific tests such as real-time PCR, virus isolation, and/or plaque reduction neutralization testing due to potential cross-reactivity of IgM ELISA with other arboviral flaviviruses [15].

The mortality rate is estimated at 10% among patients with neuroinvasive POWV infection. There are no specific antiviral treatments or vaccines available against POWV infection, and supportive care remains the mainstay of therapy [14].

## Conclusions

POWV is a potentially serious neuroinvasive infection that can cause significant neurological damage, particularly in vulnerable populations such as the elderly. With no specific antiviral treatments currently available, management relies on supportive care, early recognition, and symptom-based treatment. Prevention is key and includes using tick repellents, wearing long sleeves and pants in tick-prone areas, and performing thorough tick checks after outdoor activities. As tick populations continue to expand, the risk of POWV transmission is increasing. Raising awareness about the virus and its symptoms is essential to promote timely diagnosis, reduce unnecessary diagnostic testing, and improve patient outcomes.

## References

[1] Khan M, Beckham JD, Piquet AL, Tyler KL, Pastula DM (2019). An overview of Powassan virus disease. Neurohospitalist.

[2] (2025). Data and Maps for Powassan. https://www.cdc.gov/powassan/data-maps/index.html.

[3] Qi R, Yu H, Yu XJ (2024). Chapter 121 - Hemorrhagic fever viruses. Molecular Medical Microbiology (Third Edition).

[4] (2025). Transmission of Powassan Virus. https://www.cdc.gov/powassan/php/transmission/index.html.

[5] (2025). Treatment and Prevention of Powassan Virus Disease. https://www.cdc.gov/powassan/php/transmission/index.html.

[6] Eisen RJ, Eisen L, Ogden NH, Beard CB (2016). Linkages of weather and climate with Ixodes scapularis and Ixodes pacificus (Acari: Ixodidae), enzootic transmission of Borrelia burgdorferi, and Lyme disease in North America. J Med Entomol.

[7] Ebel GD (2010). Update on Powassan virus: emergence of a North American tick-borne flavivirus. Annu Rev Entomol.

[8] Ebel GD, Kramer LD (2004). Short report: duration of tick attachment required for transmission of powassan virus by deer ticks. Am J Trop Med Hyg.

[9] McMinn PC (1997). The molecular basis of virulence of the encephalitogenic flaviviruses. J Gen Virol.

[10] Romero JR, Simonsen KA (2008). Powassan encephalitis and Colorado tick fever. Infect Dis Clin North Am.

[11] Vahey GM, Wilson N, McDonald E (2022). Seroprevalence of Powassan virus infection in an area experiencing a cluster of disease cases: Sussex County, New Jersey, 2019. Open Forum Infect Dis.

[12] Piantadosi A, Solomon IH (2022). Powassan virus encephalitis. Infect Dis Clin North Am.

[13] Ellul M, Solomon T (2018). Acute encephalitis - diagnosis and management. Clin Med (Lond).

[14] Krow-Lucal ER, Lindsey NP, Fischer M, Hills SL (2018). Powassan virus disease in the United States, 2006-2016. Vector Borne Zoonotic Dis.

[15] Thomm AM, Schotthoefer AM, Dupuis AP 2nd (2018). Development and validation of a serologic test panel for detection of Powassan virus infection in U.S. patients residing in regions where Lyme disease is endemic. mSphere.

